# Permanent draft genome sequence of *Desulfurococcus mobilis* type strain DSM 2161, a thermoacidophilic sulfur-reducing crenarchaeon isolated from acidic hot springs of Hveravellir, Iceland

**DOI:** 10.1186/s40793-015-0128-4

**Published:** 2016-01-13

**Authors:** Dwi Susanti, Eric F. Johnson, Alla Lapidus, James Han, T. B. K. Reddy, Manoj Pilay, Natalia N. Ivanova, Victor M. Markowitz, Tanja Woyke, Nikos C. Kyrpides, Biswarup Mukhopadhyay

**Affiliations:** Department of Biochemistry, Virginia Tech, Blacksburg, VA 24061 USA; Biocomplexity Institute, Virginia Tech, Blacksburg, VA 24061 USA; Centre for Algorithmic Biotechnology, St. Petersburg State University, St. Petersburg, Russia; Algorithmic Biology Lab, St. Petersburg Academic University, St. Petersburg, Russia; US DOE Joint Genome Institute, Walnut Creek, California 94598 USA; Biological Data Management and Technology Center, Lawrence Berkeley National Laboratory, Berkeley, California USA; Department of Biology, Faculty of Science, King Abdulaziz University, Jeddah, Saudi Arabia; Department of Biological Sciences, Virginia Tech, Blacksburg, VA 24061 USA

**Keywords:** *Desulfurococcus*, Sulfur-reducing crenarchaeon, Thermophile, Acidic hot spring

## Abstract

This report presents the permanent draft genome sequence of *Desulfurococcus mobilis* type strain DSM 2161, an obligate anaerobic hyperthermophilic crenarchaeon that was isolated from acidic hot springs in Hveravellir, Iceland. *D. mobilis* utilizes peptides as carbon and energy sources and reduces elemental sulfur to H_2_S. A metabolic construction derived from the draft genome identified putative pathways for peptide degradation and sulfur respiration in this archaeon. Existence of several hydrogenase genes in the genome supported previous findings that H_2_ is produced during the growth of *D. mobilis* in the absence of sulfur. Interestingly, genes encoding glucose transport and utilization systems also exist in the *D. mobilis* genome though this archaeon does not utilize carbohydrate for growth. The draft genome of *D. mobilis* provides an additional mean for comparative genomic analysis of desulfurococci. In addition, our analysis on the Average Nucleotide Identity between *D. mobilis* and *Desulfurococcus mucosus* suggested that these two desulfurococci are two different strains of the same species.

## Introduction

*Desulfurococcus mobilis* type strain DSM 2161 was isolated from acidic hot springs in Hveravellir, Iceland [1]. This hyperthermophilic crenarchaeaon utilizes casein and peptides present in yeast extract, and tryptic digest of casein as energy and carbon source [1]. In the presence of sulfur as electron acceptor, *D. mobilis* undergoes sulfur respiration generating H_2_S and CO_2_, whereas in the absence of sulfur it performs peptide oxidation coupled to hydrogen production for regeneration of electron carriers [1, 2]. Growth in the presence of sulfur yields five times more cell density compared to that without sulfur [1].

Among known desulfurococci, *D. mobilis* is a closer relative of *Desulfurococcus mucosus* which is also a peptide degrader [1, 3]. *D. mucosus* genome was sequenced in 2011 under the *Genomic Encyclopedia of Bacteria and Archaea* program [3]. In addition to *D. mobilis* and *D. mucosus*, three desulfurococci are known, and these are *Desulfurococcus fermentans* [4, 5], *Desulfurococcus amylolyticus* [6], and *Desulfurococcus kamchatkensis* [7]. All of these organisms degrade peptides. As far as other substrates for growth, starch is used only by *Desulfurococcus fermentans* and *Desulfurococcus amylolyticus* whereas sugars can be used by *Desulfurococcus fermentans* and *Desulfurococcus kamchatkensis*. The only cellulose degrading *Desulfurococcus* is *Desulfurococcus fermentans* [4, 5]. The *Desulfurococcus fermentans* and *Desulfurococcus kamchatkensis* genomes have been sequenced by the US Department of Energy Joint Genome Institute and the Russian Academy of Sciences Centre “Bioengineering”, respectively [5, 7].

Almost all organisms that belong to the genus *Desulfurococcus* are dependent on or stimulated by sulfur [1–3, 7]. Sulfur is used as a terminal electron acceptor. The only exception is *Desulfurococcus fermentans* [4, 5] as elemental sulfur does not influence the growth of this organism and it is also the only *Desulfurococcus* species for which the growth is not inhibited by the presence of hydrogen.

The draft genome sequence of *D. mobilis* together with the complete genome sequence of *D. mucosus*, *Desulfurococcus fermentans* and *Desulfurococcus kamchatkensis* could give insight into the finer differences between peptide, starch and cellulose metabolism systems of these closely related desulfurococci leading to the discoveries of new thermophilic enzymes and pathways. Similar inquiries could be made for their differences in elemental sulfur requirements as well as their responses to the presence of H_2_ in their environment.

## Organism Information

### Classification and features

**Fig. 1:**
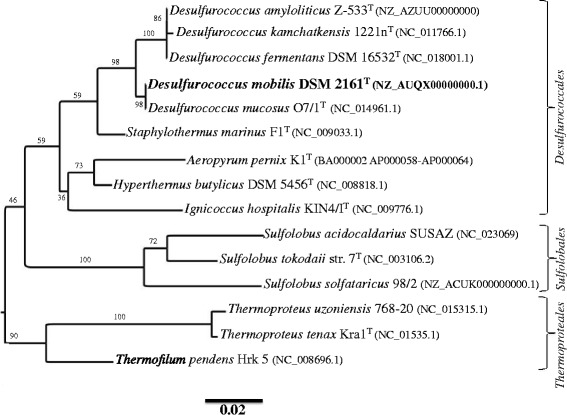
A 16S ribosomal DNA sequence-based phylogenetic tree showing the position of Desulfurococcus mobilis DSM 2161 (shown in bold) relative to other Desulfurococcus species and other organisms from Sulfolobales and Thermoproteales orders. Alignment and trimming of genes encoding 16S rRNA (aligned size of 1112 bp) were performed by the use of Muscle 3.8.31 [33] and Gblocks 0.91, respectively. The tree was constructed using Maximum Likelihood method, dnaml, in the Phylip-3.696 package [34] and viewed by the use of FigTree (http://tree.bio.ed.ac.uk/), as previously described [35]. Type strains are indicated with the superscript T. NCBI accession numbers for genome sequence are presented within parenthesis. *Methanocaldococcus jannaschii*, a euryarchaeon (not shown), was used as an outgroup [36]. Number in each branch shows a percentage of bootstrap value from 100 replicates. The bar indicates 0.02 substitutions per nucleotide position

*Desulfurococcus mobilis* belongs to the phylum *Crenarchaeota* and class of *Thermoprotei*. Within this class, three orders namely *Desulfurococcales*, *Sulfolobales* and *Thermoproteales* have been recognized. A phylogenetic tree based on 16S-ribosomal DNA sequences (Fig. 1) shows the position of *D. mobilis* relative to its neighbours. *Desulfurococcus mobilis* is closely related to *Desulfurococcus mucosus*. The value of ANI between *Desulfurococcus mobilis* and *Desulfurococcus mucosus* is 99.88. Such a high ANI value suggested that these organisms should be considered as two strains of the same species.

**Fig. 2:**
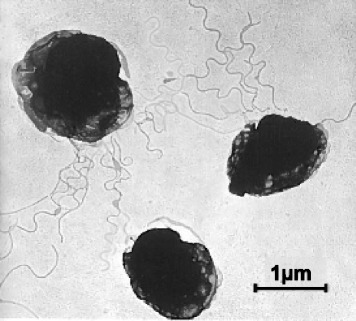
An electron micrograph of *Desulfurococcus mobilis* type strain DSM 2161 showing unipolar polytrichus archaella. The picture has been reproduced from [1] with permission

*Desulfurococcus mobilis* is a Gram-negative spherical coccus, with diameter about 0.1-1 μm [1]. Unlike *Desulfurococcus mucosus*, *Desulfurococcus mobilis* is motile [1]. The latter possesses monopolar polytrichus flagella that form bundle of 12.5 nm diameter (Fig. 2). Classification and general features of *Desulfurococcus mobilis* are shown in Table 1.

**Table 1:** Classification and general features of *Desulfurococcus mobilis* DSM 2161^T^ [37]

MIGS ID	Property	Term	Evidence code^a^
	Classification	Domain *Archaea*	TAS [38]
		Phylum *Crenarchaeota*	TAS [38]
		Class *Thermoprotei*	TAS [39]
		Order *Desulfurococcales*	TAS [40]
		Family *Desulfurococcaceae*	TAS [1]
		Genus *Desulfurococcus*	TAS [1]
		Species *Desulfurococcus mobilis*	TAS [1]
		Type strain DSM 2161/ATCC 35582	TAS [1]
	Gram stain	Negative	TAS [1]
	Cell shape	Coccus	TAS [1]
	Motility	Motile	TAS [1]
	Sporulation	Not reported	
	Temperature range	55-97 °C	TAS [1]
	Optimum temperature	85 °C	TAS [1]
	pH range; Optimum	2.2-6.5; 5.5-6.0	TAS [1]
	Carbon source	Yeast extract, bactotryptone, a tryptic-digest of casein or casein	TAS [1]
	Energy source	Chemoorganotroph	TAS [1]
	Terminal electron receptor	Elemental sulfur (favored)	TAS [1]
MIGS-6	Habitat	Free living	TAS [1]
MIGS-6.3	Salinity	Not reported	
MIGS-22	Oxygen requirement	Anaerobic	TAS [1]
MIGS-15	Biotic relationship	Not reported	
MIGS-14	Pathogenicity	Non-pathogen	NAS
MIGS-4	Geographic location	Iceland	TAS [1]
MIGS-5	Sample collection time	1981	TAS [1]
MIGS-4.1	Latitude	Not reported	
MIGS-4.2	Longitude	Not reported	
MIGS-4.3	Depth	Not reported	
MIGS-4.4	Altitude	Not reported	

^a^Evidence codes - TAS: Traceable Author Statement (i.e., a direct report exists in the literature); NAS: Non-traceable Author Statement (i.e., not directly observed for the living, isolated sample, but based on a generally accepted property for the species, or anecdotal evidence). These evidence codes are from the Gene Ontology project [41]

## Genome Sequencing Information

### Genome project history

*D. mobilis* was selected for sequencing by the Joint Genome Institute Community Sequencing Program in 2009 as part of a genome comparison project for the genus *Desulfurococcaceae*. Project information is available in the Genomes OnLine Database (Table 2) [8]. DRAFT sequencing, initial gap closure and annotation were performed by the DOE Joint Genome Institute using state-of-the-art sequencing technology [9]. The draft genome was partly assembled and annotated in 2012 and was deposited in the Integrated Microbial Genome Data Management System [10] in 2012.

**Table 2:** Project information

MIGS ID	Property	Term
MIGS 31	Finishing quality	High quality draft
MIGS 28	Libraries used	Illumina standard
MIGS 29	Sequencing platforms	Illumina
MIGS 31.2	Fold coverage	528 ×
MIGS 30	Assemblers	Velvet (version 1.1.04), ALLPATHS v. r40295
MIGS 32	Gene calling method	Prodigal
	Locus tag	YWQ
	Genome Database ID	IMG: 2513237118
	Genbank ID	AUQX00000000
	Genbank Date of Release	May 11, 2015
	GOLD ID	Gp0003960
	Bioproject	PRJNA163045
MIGS 13	Source Material Identifier	DSM 2161/ ATCC 35582
	Project relevance	Biotechnological

### Growth conditions and genomic DNA preparation

*D. mobilis* type strain DSM 2161 (ATCC 35582) was obtained from the ATCC microbiology culture collections (ATCC, Manassas, VA) and was cultivated on ATCC *Desulfurococcus* medium (medium 1558) containing Tryptone and yeast extract as the carbon and energy sources, each at final concentration of 2 g/l. Elemental sulfur and Na_2_S, at concentration of 5 g/l and 0.5 g/l, respectively, were added as electron acceptors and medium reductant.

Chromosomal DNA was isolated using a method as described previously [11]. Briefly, cell pellet of *D. mobilis* was resuspended in TE buffer (10 mM Tris–HCl, 1 mM EDTA, pH 8.0). Proteinase K, EDTA and Sodium dodecyl sulfate (SDS) were added to the suspension at the final concentrations of 100 μg/ml, 5 mM, and 0.5 %, respectively. The mixture was then incubated at 55 °C for one hour. An equal volume of a mixture containing phenol, chloroform, and isoamylalcohol (25:24:1, v/v/v) was added to the cell lysate and the resulting emulsion was centrifuged at 10,000 xg for 30 min. To the recovered aqueous layer containing DNA, an equal volume of a mixture of chloroform, and isoamylalcohol (24:1, v/v) was added and then the combination was centrifuged at 10,000 × g for 30 min. To the aqueous solution recovered from this step, sodium acetate-acetic acid buffer, pH 5.3 at a final concentration of 15 mM and an equal volume of isopropanol were added to precipitate chromosomal DNA. DNA was pelleted by centrifugation at 15,000 × g for 30 min and then washed with ice-cold 70 % ethanol for three times, air dried and suspended in TE buffer.

### Genome sequencing and assembly

The draft genome of *Desulfurococcus mobilis* type strain DSM 2161 was generated at the DOE Joint genome Institute using the Illumina technology [12]. An Illumina standard shotgun library was constructed and sequenced using the Illumina platform which generated 17,620,486 reads of 150 bp. All general aspects of library construction and sequencing performed at the JGI can be found at JGI website. All raw Illumina sequence data was passed through DUK, a filtering program developed at JGI (Mingkun, L., Copeland, A. and Han, J., unpublished program), which removes known Illumina sequencing and library preparation artifacts. Following steps were then performed for assembly: (1) filtered Illumina reads were assembled using Velvet [13], (2) 1–3 kb simulated paired end reads were created from Velvet contigs using wgsim [14], (3) Illumina reads were assembled with simulated read pairs using Allpaths–LG [15, 16]. Parameters for assembly steps were: 1) Velvet (velveth: 63 –shortPaired and velvetg: −very clean yes –exportFiltered yes –min contig lgth 500 –scaffolding no –cov cutoff 10) 2) wgsim (−e 0 –1 100 –2 100 –r 0 –R 0 –X 0) 3) Allpaths–LG (PrepareAllpathsInputs: PHRED 64 = 1 PLOIDY = 1 FRAG COVERAGE = 125 JUMP COVERAGE = 25 LONG JUMP COV = 50, RunAllpathsLG: THREADS = 8 RUN = std shredpairs TARGETS = standard VAPI WARN ONLY = True OVERWRITE = True). The final draft assembly contained 58 contigs.

### Genome annotation

Genes were identified using Prodigal [17] as part of the JGI’s microbial genome annotation pipeline [17]. The predicted coding sequences were translated and used to search the National Center for Biotechnology Information nonredundant database, UniProt, TIGR-Fam, Pfam, PRIAM, KEGG, COG, and InterPro databases. Identification of RNA genes were carried out by using HMMER 3.0rc1 [18] (rRNAs) and tRNAscan-SE 1.23 (tRNAs) [19]. Other non-coding genes were predicted using INFERNAL 1.0.2 [20]. Additional annotation was performed within the Integrated Microbial Genomes - Expert Review platform [21]. CRISPR elements were detected using CRT [22] and PILER-CR [23].

## Genome Properties

The draft genome of *D. mobilis* consists of a 1,198,142 bp chromosome with 52.89 % GC content. It contains 1,277 protein coding genes, and 54 ribosomal RNA genes that encode 1, 2, 41, and 10 of 16S-, 23S-ribosomal RNA, tRNA and other RNAs, respectively. Tables 3 and 4 present genome statistics, and distribution of genes into COG categories, respectively.

**Table 3:** Genome statistics

Attribute	Value	% of total
Genome size (bp)	1,198,142	100.00
DNA coding (bp)	1,084,053	90.48
DNA G + C (bp)	633,652	52.89
DNA scaffolds	58	100.00
Total genes	1,331	100.00
Protein-coding genes	1,277	95.94
RNA genes	54	4.06
Pseudo genes	NA	NA
Genes in internal clusters	89	6.69
Genes with function prediction	970	72.88
Genes assigned to COGs	843	63.34
Genes with Pfam domains	948	71.22
Genes with signal peptides	10	0.75
Genes with transmembrane helices	218	16.38
CRISPR repeats	5	-

**Table 4:** Number of genes associated with general COG functional categories

Code	Value	%age	Description
J	176	13.78	Translation, ribosomal structure and biogenesis
A	1	0.08	RNA processing and modification
K	41	3.13	Transcription
L	40	3.6	Replication, recombination and repair
B	1	0.08	Chromatin structure and dynamics
D	7	0.47	Cell cycle control, cell division, and chromosome partitioning
V	18	0.55	Defense mechanisms
T	16	0.78	Signal transduction mechanisms
M	30	1.96	Cell wall/membrane biogenesis
N	4	0.31	Cell motility
U	9	0.78	Intracellular trafficking and secretion
O	43	3.21	Posttranslational modification, protein turnover, chaperones
C	76	6.03	Energy production and conversion
G	44	3.13	Carbohydrate transport and metabolism
E	62	4.86	Amino acid transport and metabolism
F	40	2.74	Nucleotide transport and metabolism
H	59	3.29	Coenzyme transport and metabolism
I	17	0.86	Lipid transport and metabolism
P	66	5.32	Inorganic ion transport and metabolism
Q	2	0.23	Secondary metabolites biosynthesis, transport and catabolism
R	102	10.73	General function prediction only
S	47	6.81	Function unknown
-	488	38.21	Not in COGs

The total is based on the total number of protein coding genes in the annotated genome

## Insights from the Genome Sequence

A metabolic construction derived from the draft genome indicates that a *Pyrococcus furiosus*-type peptide degradation pathway operates in *D. mobilis* [24]. Peptides likely enter the cell via peptide/amino acid transporters that are encoded by YWQDRAFT_00113, 00114, 00115, and 00118. Once inside the cell, peptides are catabolized into amino acids by peptidases. A total of 10 peptidases were identified in the draft genome of *D. mobilis*. An example is YWQDRAFT_00964 that is a homolog of pyroglutamyl peptidase of *Desulfurococcus fermentans* (Desfe_1254) with e-value of 2e-63. The resulting amino acids are then converted into their respective keto-acids in reactions catalyzed by transaminases (YWQDRAFT0500, 00632, 00843, 00124). These keto-acids are catabolized further into acyl-CoA by several putative keto-acid:ferredoxin oxidoreductase such as indole pyruvate ferredoxin oxidoreductase (YWQDRAFT_00457 and 00458), aldehyde ferredoxin oxidoreductase (YWQDRAFT_00049 and 00586), and pyruvate ferredoxin oxidoreductase (YWQDRAFT_00252, 00251, 00253, 00254). Then ATP generation occurs via the acetyl-CoA synthetase reaction (YWQDRAFT_00758).

In the presence of sulfur, electrons generated from peptide oxidation are transferred into sulfur via a sulfur reductase (YWQDRAFT_00031), a cytoplasmic protein with high similarity to NADPH-dependent polysulfide reductase of *Desulfurococcus kamchatkensis* (ORF Dkam_0441) [7] and sulfide dehydrogenase of *Pyrococcus furiosus* that is composed of two subunits, A and B (ORF PF1327-28) [25]. This process generates H_2_S and a proton motive force and the latter helps to synthesize ATP via ATPase (YWQDRAFT_00542).

Genome analysis also reveals genes encoding putative Ni-Fe hydrogenases that were found in three hydrogenase clusters (YWQDRAFT_01235-01241; 01256–64, 01282–01285; and 00877–00866). This finding explains previous observation that during growth in the absence of elemental sulfur *D. mobilis* produces hydrogen to dispose off electrons originating from peptide degradations [1, 2].

Similarly, enzymes for converting acetyl-CoA to glucose-6-phosphate via gluconeogenesis pathways and for glycogen synthesis were found. Key enzymes for gluconeogenesis were phosphoenolpyruvate synthase (YWQDRAFT_00160) and 1,6-fructosebisphosphatase (YWQDRAFT_00288). The ORF for a characteristic enzyme for glycogen synthesis, glycogen synthase (YWQDRAFT_00470), was also found.

Although *D. mobilis* does not use sugars as carbon source [1], genes for two sugar transporters (YWQDRAFT_00575-76) were found in the genome. Similarly, key enzymes of the modified Emden-Meyerhof pathway [26], namely glyceraldehyde-3-phosphate ferredoxin oxidoreductase/GAPOR (YWQDRAFT_00049 and 00586) that converts glyceraldehyde-3-phosphate into 3-phosphoglycerate and pyruvate kinase (YWQDRAFT_00285) that dephosphorylates phosphoenolpyruvate to form pyruvate were detected in the genome. The two GAPOR homologs show 38 % and 21 % identity with the same enzymes of *Methanococcus maripaludis* [27], while the pyruvate kinase is similar to that of *Thermoproteus tenax* showing 36 % of identity [28]. In accordance, we hypothesize that *D. mobilis* utilizes carbohydrates at least as co-substrates.

As expected, *D. mobilis* genome carries *fla*I (YWQDRAFT_00614) that encodes a type IV secretory pathway/VirB11 component, which would be involved in the biogenesis of archaeal flagellum (archaellum) [29–31]. However, genes encoding known archaeal and bacterial flagellins are absent in the draft genome [32]. Since the genome sequence of *D. mobilis* is at a draft stage and approximately 100 kb of genome sequence is missing, as estimated from the average size of other desulfurococci, it is possible that the flagella structural genes are located in the missing regions. Therefore, a complete genome sequence of *D. mobilis* is needed to rule out the possibility of a novel flagella system in this organism.

## Conclusions

This study presents the genome sequence and metabolic reconstruction of *Desulfurococcus mobilis* type strain DSM 2161. The genome revealed three hydrogenase clusters that are likely responsible for electron disposal during growth in the absence of sulfur. The presence of genes encoding sugar transporters and key enzymes of the Embden Meyerhoff pathway raises the possibility of sugar utilization in *D. mobilis*. The near 100 % value of Average Nucleotide Identity for this archaeon and its close relative *D. mucosus* indicated that these organisms are very similar and reclassification of these two desulfurococci into two strains is suggested.

## Abbreviations

TIGR: The Institute for Genome Research
Pfam: Protein family database
PRIAM: Profils pour l’Identification Automatique du Métabolisme
KEGG: Kyoto Encyclopedia of Genes and Genomes
COG: Clusters of Orthologous Groups of proteins
CSP: Community Sequencing Program
ANI: Average Nucleotide Identity
